# Similar sequences but dissimilar biological functions of GDF11 and myostatin

**DOI:** 10.1038/s12276-020-00516-4

**Published:** 2020-10-19

**Authors:** Joonho Suh, Yun-Sil Lee

**Affiliations:** Department of Molecular Genetics & Dental Pharmacology, School of Dentistry and Dental Research Institute, Seoul National University, Seoul, Republic of Korea

**Keywords:** Growth factor signalling, Transforming growth factor beta, Evolutionary biology

## Abstract

Growth differentiation factor 11 (GDF11) and myostatin (MSTN) are closely related TGFβ family members that are often believed to serve similar functions due to their high homology. However, genetic studies in animals provide clear evidence that they perform distinct roles. While the loss of *Mstn* leads to hypermuscularity, the deletion of *Gdf11* results in abnormal skeletal patterning and organ development. The perinatal lethality of *Gdf11*-null mice, which contrasts with the long-term viability of *Mstn*-null mice, has led most research to focus on utilizing recombinant GDF11 proteins to investigate the postnatal functions of GDF11. However, the reported outcomes of the exogenous application of recombinant GDF11 proteins are controversial partly because of the different sources and qualities of recombinant GDF11 used and because recombinant GDF11 and MSTN proteins are nearly indistinguishable due to their similar structural and biochemical properties. Here, we analyze the similarities and differences between GDF11 and MSTN from an evolutionary point of view and summarize the current understanding of the biological processing, signaling, and physiological functions of GDF11 and MSTN. Finally, we discuss the potential use of recombinant GDF11 as a therapeutic option for a wide range of medical conditions and the possible adverse effects of GDF11 inhibition mediated by MSTN inhibitors.

## Growth factors: untangling twin proteins

A growth factor protein called GDF11 has potential for rejuvenation of heart, brain, and muscle tissues, but first its relationship with a twin growth factor (MSTN) must be unraveled. Although GDF11 and MSTN are almost identical in amino acid sequence, MSTN controls growth of skeletal muscles, and GDF11 regulates bone and organ patterning. To untangle what differentiates these proteins, Yun-Sil Lee and Joonho Suh at Seoul National University, South Korea, reviewed their evolution and function. They report that GDF11 and MSTN are the result of duplication of an original gene and have evolved to perform different roles. They cover some of the beneficial effects reported with GDF11 supplementation, such as tissue rejuvenation and increased lifespan, but caution that negative effects have been reported. Further investigation may illuminate growth and development processes, and therapeutic potential.

## Introduction

Cytokines of the transforming growth factor β (TGFβ) family, including activins, growth differentiation factors (GDFs), bone morphogenetic proteins (BMPs), and TGFβs, have been extensively implicated in the regulation of developmental patterning, cellular proliferation and differentiation, and the maintenance of tissue homeostasis^1^. Among the TGFβ family members, there are two highly homologous proteins, GDF11 and myostatin (MSTN), which share 89% sequence identity in their mature form but exhibit distinct endogenous functions. While *Gdf11* is expressed broadly in numerous tissues, *Mstn* is expressed primarily in skeletal muscle^2–4^. The functional divergence of GDF11 and MSTN is indicated by the fact that their mutation in animals leads to the development of largely dissimilar features. For instance, while the genetic deficiency of *MSTN* leads to a hypermuscular phenotype in various species^4–8^, homozygous deletion of *Gdf11* generates defects in axial skeletal patterning and organ development in mice^9^. However, unlike the relatively consistent reports of the function of MSTN in suppressing skeletal muscle growth, the reports of GDF11 function, particularly those examining the postnatal role of GDF11, remain highly controversial. One of the main reasons for this controversy lies in the fact that *Gdf11-*null mice, unlike *Mstn*-null mice, show perinatal lethality^9^, leading most studies to utilize recombinant proteins that cannot fully recapitulate the complex endogenous functions of GDF11. Importantly, in contrast to studies that utilized recombinant GDF11 or MSTN proteins, those that applied genetic knockdown, knockout, or conditional knockout techniques revealed relatively unvarying results despite their being fewer in number, and most have reported the positive roles of GDF11 and the negative roles of MSTN in the regulation of the development of various tissues. In this review, we first present the similarities and differences between GDF11 and MSTN from an evolutionary point of view and summarize the insights obtained to date regarding the biological processing, signaling mechanisms, and physiological functions of GDF11 and MSTN during development, adulthood, and aging. We also discuss the potential of recombinant GDF11 protein as a therapeutic option for various clinical conditions and the possible adverse effects of GDF11 inhibition mediated by MSTN inhibitors.

## Evolution and biology of GDF11 and MSTN

### Evolutionary analysis of GDF11 and MSTN

**Fig. 1:**
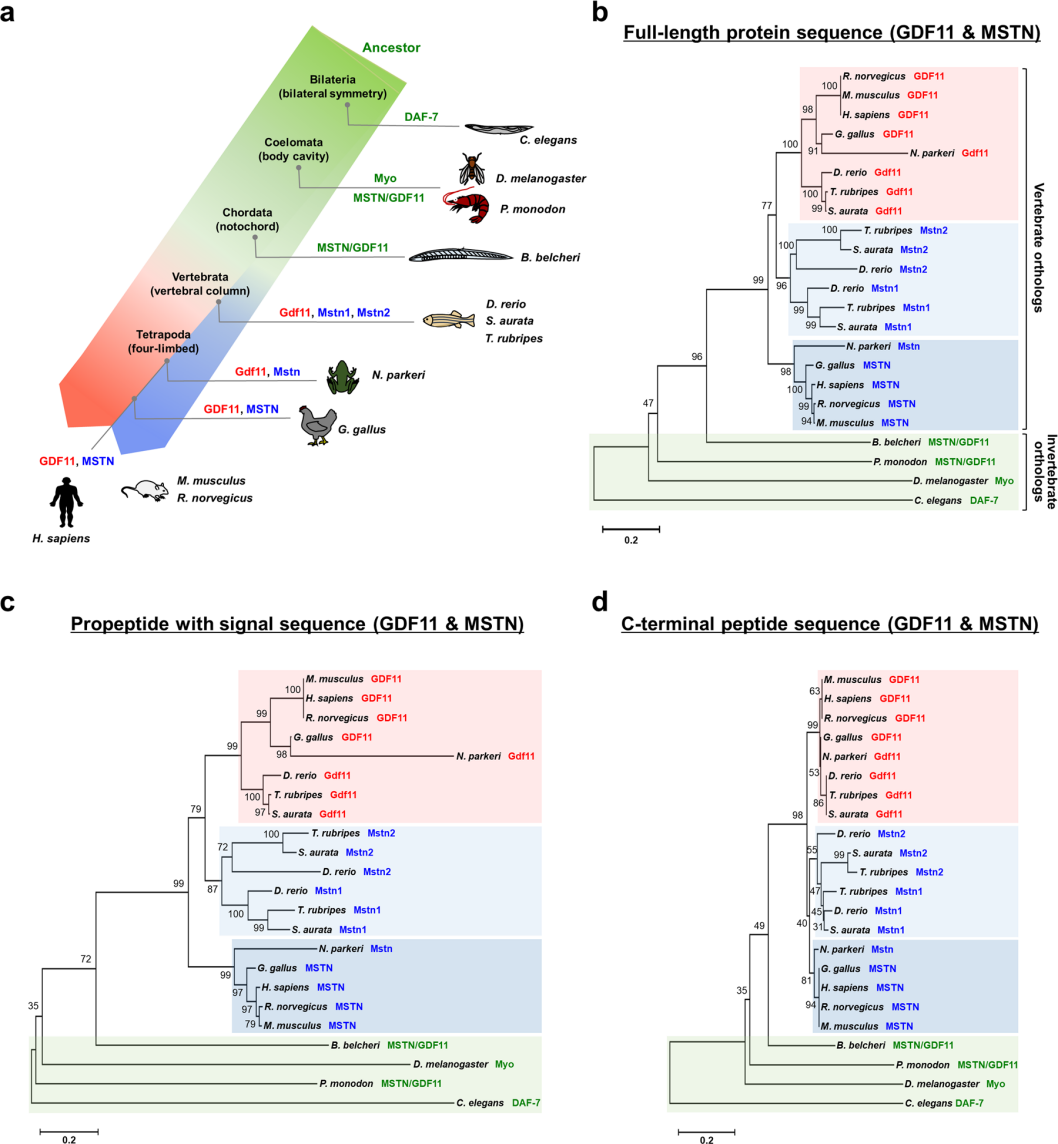
Evolutionary relationships among vertebrate GDF11, MSTN, and invertebrate MSTN/GDF11. **a** Simplified diagram representing the phylogenetic analysis of GDF11, MSTN, and invertebrate MSTN/GDF11. Note that the gene duplication event generating *GDF11* and *MSTN* occurred at the time of the emergence of vertebrates. **b** Phylogenetic tree generated by full-length protein sequence comparison. **c** Phylogenetic tree generated by N-terminal (propeptide with signal peptide) sequence comparison. **d** Phylogenetic tree generated by C-terminal peptide sequence comparison. Multiple sequence alignments were performed using MEGA X software^127^ and the MUSCLE (multiple sequence comparison by log-expectation) algorithm^128^. All phylogenetic trees were constructed using MEGA X software by applying the neighbor-joining method, bootstrap method (1000 replicates), and Jones−Taylor−Thornton model. Gaps and missing data were treated as complete deletions. The numbers at the tree nodes indicate the percentage bootstrap values. Scale bars represent the number of substitutions per site.

The remarkable sequence similarity between GDF11 and MSTN has led to the assumption that they were derived from the same ancestral gene through gene duplication. Indeed, analysis of multiple invertebrate species revealed that they harbor a single homologous gene corresponding to *GDF11* and *MSTN*^10^. For instance, in *Caenorhabditis elegans*, *daf-7* was shown to encode a homolog of *GDF11* and *MSTN*, while in fruit flies (*Drosophila melanogaster*), myoglianin (Myo) was found to exhibit the highest sequence homology to GDF11 and MSTN^10–13^. An important question that arose from these identifications was whether the divergence of *GDF11* and *MSTN* occurred at the time of the emergence of vertebrates. To provide an explanation, a phylogenetic study was conducted in various invertebrate and vertebrate species, and importantly, the amphioxus (*Branchiostoma belcheri*)^14^, which is an invertebrate known to be the closest relative of the vertebrates, was included in the analysis (Fig. 1a and Table 1). Additionally, the amino acid sequences of the full-length protein, the propeptide with the signal peptide, and C-terminal peptide were separately compared (Fig. 1b−d). All phylogenetic trees demonstrated a clear separation between the GDF11 and MSTN clusters that appeared after the divergence of vertebrates from the amphioxus, confirming that the gene duplication event occurred at the time when vertebrates and invertebrates split (Fig. 1b−d). Notably, unlike the single isoform of the *MSTN* gene observed in mammals, two isoforms of the *mstn* gene have been detected in fish^10^. The reason for and functional significance of the divergence of the two *mstn* genes in fish remains to be clarified. Interestingly, many of the reported functions of the invertebrate MSTN/GDF11 protein are very different from the well-established suppressive role of vertebrate MSTN in the development of multiple tissues, and the broad expression pattern of the ancestral protein more closely resembles the expression pattern of vertebrate GDF11 ^11,13,15–19^. These observations imply that MSTN most likely emerged from the ancestral gene to allow more specific control of skeletal muscle growth in vertebrates, although the relatively small amount of information available on the function of invertebrate MSTN/GDF11 limits further interpretation. The reported physiological roles of the ancestral protein in invertebrates will be discussed in more detail later.

**Table 1:** List of proteins, species, and accession numbers used for phylogenetic analysis.

Growth factor	Species	Common name	Accession no.
DAF-7^a^	*Caenorhabditis elegans*	Nematode worm	AAC47389
Myo^a^	*Drosophila melanogaster*	Common fruit fly	NP_726604
MSTN/GDF11^a^	*Penaeus monodon*	Asian tiger shrimp	ADO34177
	*Branchiostoma belcheri*	Amphioxus	ABS59067
Gdf11	*Takifugu rubripes*	Japanese puffer fish	XP_029682178
	*Danio rerio*	Zebrafish	NP_998140
	*Sparus aurata*	Gilthead sea bream	XP_030277152
	*Nanorana parkeri*	High Himalaya frog	XP_018417520
GDF11	*Gallus gallus*	Red junglefowl	XP_025001403
	*Mus musculus*	Mouse	NP_034402
	*Rattus norvegicus*	Rat	NP_058899
	*Homo sapiens*	Human	NP_005802
Mstn1	*Takifugu rubripes*	Japanese puffer fish	AAR88255
	*Danio rerio*	Zebrafish	AAB86693
	*Sparus aurata*	Gilthead sea bream	AAK53545
Mstn2	*Takifugu rubripes*	Japanese puffer fish	AAR88254
	*Danio rerio*	Zebrafish	Q68IN2
	*Sparus aurata*	Gilthead sea bream	AAL05943
Mstn	*Nanorana parkeri*	High Himalaya frog	XP_018425732
MSTN	*Gallus gallus*	Red junglefowl	AAR18244
	*Mus musculus*	Mouse	NP_034964
	*Rattus norvegicus*	Rat	NP_062024
	*Homo sapiens*	Human	NP_005250

*Myo* myoglianin, *GDF11* growth differentiation factor 11, *MSTN* myostatin.

^a^Represents growth factors present in invertebrates. Note that GDF11 and MSTN have common ancestors in invertebrates.

### Proteolytic processing of GDF11 and MSTN

Both GDF11 and MSTN, like the other members of the TGF-β family, are initially synthesized as precursor proteins and are subsequently cleaved by proteases to produce biologically active mature ligands. More specifically, following the removal of the signal peptides by signal peptidases, furin-like proteases recognize and cleave the conserved RSRR residues of GDF11 and MSTN, generating N-terminal propeptides and C-terminal mature peptides^20^. The different types of furin-like proprotein convertases and their substrates are listed in Table 2. The proprotein convertase PC5/6 was demonstrated to specifically cleave GDF11 by recognizing the RSRR↓N cleavage motif, which is not present in MSTN^21^. Accordingly, mice deficient in PC5/6 were shown to phenocopy *Gdf11*-null mice by exhibiting anterior homeotic transformations of the vertebrae, the lack of a tail, kidney agenesis, and retarded ossification^21^. After the cleavage of the RSRR site by a furin-like protease, the propeptide and mature peptide remain noncovalently associated with each other, forming a latent complex that is unable to bind receptors. However, a recent study showed that the latent MSTN complex can also become capable of binding receptors after being exposed to acidic conditions. Exposure to acidic conditions led to a conformational change of the latent MSTN complex and stimulated it to become in a triggered state, in which the pro- and mature domains still remain associated but were capable of signaling^22^. The fact that MSTN can exist in both fully latent and triggered states further demonstrates the complexity of its activation mechanism. Nonetheless, to achieve full signaling activity, both the latent GDF11 and MSTN complexes require additional cleavage of the N-terminal propeptides by BMP1/tolloid (TLD)-like metalloproteinases, which dissociate the propeptides from the mature C-terminal dimers, thus freeing the ligands for receptor binding (Table 3)^20^. Mature dimers can also be inhibited by the addition of propeptides both in vitro and in vivo^23^.

**Table 2:** Types and characteristics of proprotein convertases.

Proprotein convertase	Cleavage site	Expression pattern	Localization	Substrates	Mutant phenotype
PC1/3	(K/R)R↓	Neuroendocrine cells	Secretory granule	GHRH, ACTH, insulin, GLPs, substrate overlap with PC2	Dwarfism^131,132^
PC2	RX(K/R)R↓	Neuroendocrine cells	Secretory granule	Glucagon, insulin, β-endorphin, α-MSH, substrate overlap with PC1/3	Retarded growth, hypoglycemia^132,133^
Furin	RX(K/R)R↓	Ubiquitous	TGN, cell surface, ECM	Growth factors (TGFβs, MSTN, GDF11, Inhibins, BMPs, Nodal, Lefty), insulin receptor, MMPs, viral glycoproteins, bacterial toxins	Embryonic death, impaired axial rotation^132,134^
PC4	RX(K/R)R↓	Germ cells	Cell surface	IGF2, PACAP	Reduced fertility^132,135^
PC5/6	RX(K/R)R↓	Widespread	TGN, cell surface, ECM	Growth factors (GDF11, BMP2), substrate overlap with furin	Phenotype of *Gdf11*-null mice (reduced bone development and skeletal patterning defects)^21,132^
PACE4	RX(K/R)R↓	Widespread	TGN, cell surface, ECM	Growth factors (Nodal and Lefty), substrate overlap with furin	Defects in anterior CNS patterning and left-right axis formation, craniofacial malformation^132,136^
PC7	RX(K/R)R↓	Ubiquitous	TGN, cell surface	Partial substrate overlap with furin	Impaired cognitive performance^132,137,138^
SKI-1/S1P	RX(L/V/I)X↓	Ubiquitous	*cis*/*medial* Golgi, cell surface	Transcription factors (SREBPs, ATF6, CREBs), GlcNAc-1-phosphotransferase, viral glycoproteins	Embryonic death, lack of epiblast formation^132,139^
PCSK9	(V/I/L)FAQ↓	Liver, intestine, kidney	Cell surface, ECM	PCSK9, interaction with LDLR	Hypocholesterolemia^132^

*ACTH* adrenocorticotropic hormone, *α-MSH* α-melanocyte-stimulating hormone, *ATF6* activating transcription factor 6, *BMP* bone morphogenetic protein, *CREB* cyclic AMP-responsive element-binding protein, *ECM* extracellular matrix, *GDF11* growth differentiation factor 11, *GHRH* growth hormone-releasing hormone, *GlcNAc* *N*-acetylglucosamine, *GLP* glucagon-like peptide, *IGF2* insulin-like growth factor 2, *LDLR* low-density lipoprotein receptor, *MMP* matrix metalloproteinase, *MSTN* myostatin, *PACAP* pituitary adenylyl cyclase-activating peptide, *PACE4* paired basic amino acid-cleaving enzyme 4, *PCSK9* proprotein convertase subtilisin kexin 9, *SKI-1* subtilisin kexin isozyme 1, *SREBP* sterol regulatory element-binding protein, *TGFβ* transforming factor-β, *TGN* *trans*-Golgi network.

**Table 3:** Types and characteristics of BMP-1/tolloid-like metalloproteinases.

Proprotein convertase	Cleavage site	Expression pattern	Localization	Substrates	Mutant phenotype
BMP1	↓D	Widespread	TGN, ECM	MSTN, GDF11, Chordin, Decorin, LTBP1, DMP1, DSP-PP, Procollagen I-III, prolysyl oxidase, Prolaminin 5, Probiglycan	Perinatal death with failure of ventral body wall closure (knockout mice), osteogenesis imperfecta (conditional knockout mice)^140–142^
mTLD (BMP1 isoform)	↓D	Widespread	TGN, ECM	Substrate overlap with BMP1 (mTLD is a less efficient C-proteinase and cannot cleave chordin)	Equal to those of BMP1^140,141^
TLL1	↓D	Widespread	ECM	MSTN, GDF11, Chordin, Procollagen I, II, and VII, Lysyl pro-oxidase, Osteoglycine, Decorin, Probiglycan, Perlecan	Embryonic death due to cardiovascular defects^143,144^
TLL2	↓D	Skeletal muscle	ECM	MSTN, GDF11	Slightly increased muscle weight^145,146^

*BMP1* bone morphogenetic protein 1, *DMP1* dentin matrix acidic phosphoprotein 1, *DSPP* dentin sialophosphoprotein, *ECM* extracellular matrix, *GDF11* growth differentiation factor 11, *LTBP1* latent transforming growth factor beta-binding protein 1, *MSTN* myostatin, *mTLD* mammalian tolloid, *TGN* *trans*-Golgi network, *TLL* tolloid-like.

**Fig. 2:**
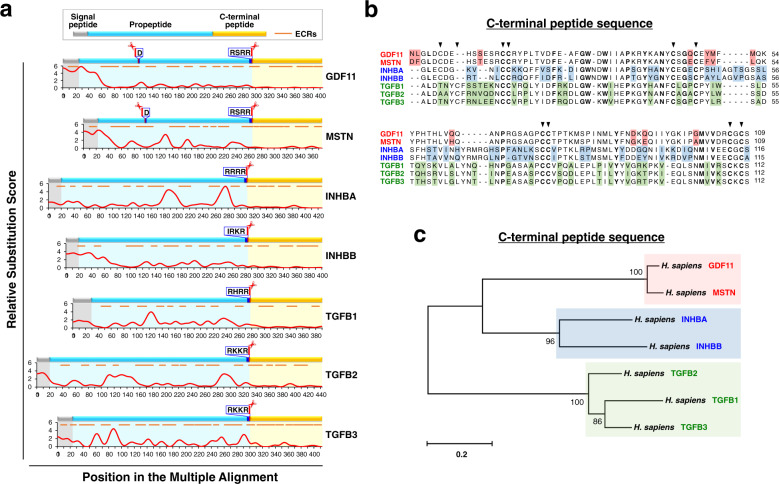
Analysis of evolutionarily constrained regions (ECRs) of GDF11, MSTN, activins, and TGFβs. **a** Profiles of the relative rates of amino acid substitution and ECRs generated using Aminode^24^. Raw data are publicly available on the Aminode website. The analyzed vertebrates are listed in Supplementary Table S1. Gray, blue, and yellow represent the signal peptide, propeptide, and C-terminal peptide, respectively. Cleavage sites (RXXR) recognized by furin-like proteases are labeled with scissors. GDF11 and MSTN contain additional cleavage sites (D) recognized by BMP1/TLD-like metalloproteinases. **b** Multiple sequence alignments of C-terminal peptides of human GDF11, MSTN, INHBA, INBB, TGFB1, TGFB2, and TGFB3 performed using MEGA X software^127^ and the MUSCLE algorithm^128^. The conserved cysteines are marked with triangles. Residues that differ between GDF11 and MSTN, between INHBA and INHBB, and among TGFBs are highlighted in red, blue, and green, respectively. **c** Phylogenetic tree generated by human C-terminal peptide sequence comparison. The tree was constructed based on the method described in Fig. 1. The scale bar represents the number of substitutions per site.

To examine the rates of the evolutionary changes of the residues of GDF11 and MSTN, we utilized a recently developed webtool, Aminode^24^, and analyzed the evolutionarily constrained regions (ECRs) of the proteins (Fig. 2a and Supplementary Table S1). As expected, the mature domains of GDF11, MSTN, activins, and TGF-βs were remarkably well-conserved among vertebrate species, displaying extremely low rates of amino acid substitution in most positions (Fig. 2a). Surprisingly, only GDF11 presented a striking degree of sequence conservation in the prodomain, emphasizing the functional significance of this region (Fig. 2a). In fact, while GDF11 and MSTN share 89% amino acid sequence identity in their mature domains, which differ by only 11 residues (Fig. 2b, c), their prodomains share only 48% amino acid sequence identity. This suggests the strong possibility that GDF11 prodomains may be associated with distinct and crucial extracellular regulatory mechanisms and biological functions that are not observed for the prodomains of MSTN, which warrants further investigation that may uncover significant differences that were previously unnoticed for the mature ligands.

### Molecular mechanisms of GDF11 and MSTN signaling

**Fig. 3:**
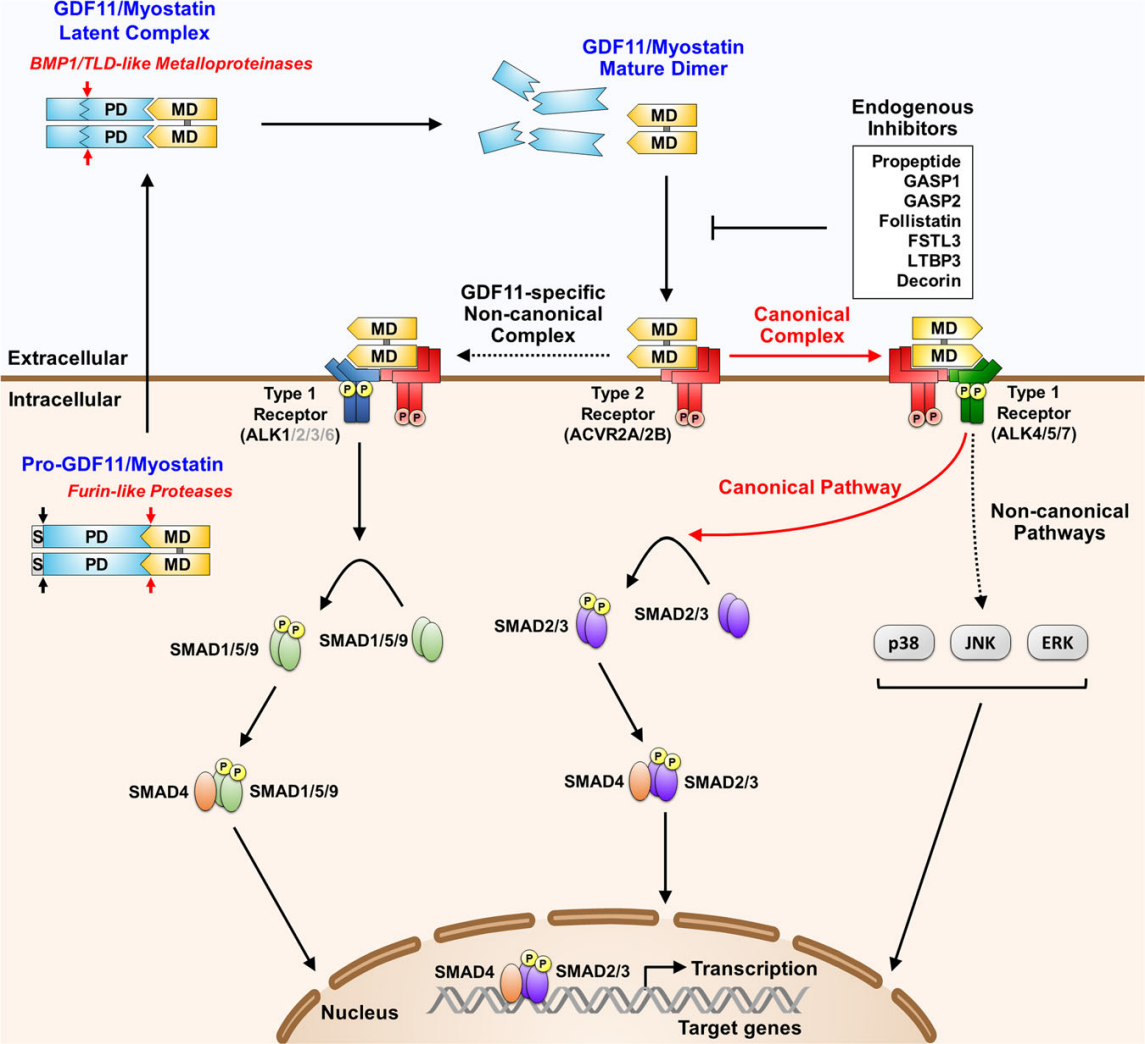
Processing, extracellular regulation, and signaling mechanisms of GDF11 and MSTN. GDF11 and MSTN are initially synthesized as precursor proteins that undergo proteolytic processing to generate biologically active mature dimers. After removal of the signal peptide (S) by a signal peptidase, pro-GDF11/MSTN is cleaved by a furin-like protease to produce a latent complex, in which the mature domain (MD) is noncovalently associated with the prodomain (PD). The latent complex is additionally cleaved by a BMP1/TLD-like metalloproteinase to generate the mature, disulfide-linked dimer (marked in dark gray) that elicits signal transduction. The latent complex has been shown to be capable of existing in a triggered state, allowing it to initiate signaling events^22^. The mature GDF11/MSTN ligand binds to activin type 2 receptors (ACVR2A/2B) that subsequently recruit activin type 1 receptors (ALK4/5/7) to signal through the canonical SMAD2/3 pathway. Activation of noncanonical pathways, including ERK, JNK, and p38 MAPK, has also been reported^20^. In addition, GDF11 has been demonstrated to activate the SMAD1/5/9 pathway in endothelial cells and osteoblasts^26,27,129,130^. Dotted lines with an arrowhead indicate noncanonical pathways, and solid lines with an arrowhead indicate canonical pathways.

The mature GDF11 and MSTN ligands first bind to activin type 2 receptors (ACVR2A or ACVR2B) and subsequently recruit type 1 receptors, activin receptor-like kinase 4 (ALK4) or ALK5 to form a heteromeric receptor complex to elicit downstream signaling via phosphorylation of SMAD2 and/or SMAD3 (Fig. 3)^20^. Both GDF11 and MSTN were recently revealed to be capable of also recruiting ALK7, while GDF11 signaled more potently than MSTN through this receptor^25^. Structural analysis also demonstrated that mature GDF11 and MSTN share identical type 2 receptor-binding residues but exhibit differences in the prehelix loop and wrist helix of the type 1 receptor-binding site^20^. Indeed, GDF11 was shown to signal more effectively and induce a greater SMAD3-dependent signal through all type 1 receptors than MSTN, and substitution of the residues in the wrist helix of the MSTN type 1 interface with those of GDF11 significantly enhanced the potency of MSTN by improving the stability of the interaction between the prehelix loop and wrist helix^25^. In addition to stimulating SMAD2/3 phosphorylation, recent studies described that GDF11 can strongly activate SMAD1/5/9 phosphorylation in endothelial cells and osteoblasts to promote their proliferation and differentiation, respectively^26,27^. GDF11 was shown to utilize ALK1 receptors to elicit signal transduction through SMAD1/5/9 phosphorylation, which was effectively suppressed by siRNA-mediated knockdown of GDF11^27^. Via another layer of complexity, GDF11 and MSTN may signal through noncanonical pathways by activating other non-SMAD proteins, such as p38 MAPK, ERK, and JNK^20^.

The activities of mature GDF11 and MSTN are tightly modulated by different extracellular binding proteins, including follistatin (FST), follistatin-like 3 (FSTL3/FLRG), growth and differentiation factor-associated serum protein 1 (GASP1), GASP2, latent TGF-β binding protein 3 (LTBP3), and decorin^20^. In contrast to the FST-like proteins that antagonize a variety of molecules of the TGF-β family, GASP1 and GASP2 more selectively inhibit mature GDF11 and MSTN^28^. While GASP1 was shown to more potently bind MSTN/GDF11 than GASP2 in vitro^29^, GASP2 was shown to more specifically regulate GDF11 based on the similarity of the embryonic expression patterns of *Gasp2* and *Gdf11* and the phenotype of *Gasp2* knockout mice, which exhibited posterior homeotic transformations indicative of GDF11 overactivity^30^. Recently, Parente et al.^31^ demonstrated that transgenic mice ubiquitously overexpressing GASP1 and GASP2 present distinct phenotypes with contrasting expression patterns of *Gdf11* and *Mstn*. The study illustrated that *Mstn* expression was significantly upregulated in GASP1-overexpressing mice, which showed an increase in low oxidative muscle fibers and impaired metabolic homeostasis, but only *Gdf11* but not *Mstn* expression was significantly elevated in GASP2-overexpressing mice, which exhibited an increase in fast glycolytic muscle fibers without metabolic defects^31^. These results provide evidence that distinct extracellular regulatory mechanisms and endogenous functions are associated with GDF11 and MSTN.

Both GDF11 and MSTN circulate in the blood, and maternal deficiency of MSTN was shown to stimulate additional muscle growth in *Mstn*-knockout pups^32^, implying that GDF11 and MSTN may function as endocrine signaling molecules. However, our previous findings in mosaic mice in which *Mstn* was deleted exclusively in posteriorly located muscles highlighted the important paracrine function of MSTN in addition to its endocrine action in regulating muscle mass^33^. Furthermore, whether circulating GDF11 levels have physiological relevance was formerly questioned based on a result showing that the molar concentration of circulating GDF11 was approximately 500 times less than that of MSTN^34^. Because GDF11 and MSTN circulate mostly in inactive, latent forms^35^, their local activation patterns and localization of antagonists may largely contribute to distinct physiological effects of GDF11 and MSTN. Therefore, due to the complex modes of action of GDF11 and MSTN, considerable caution is required for the interpretation of the results of tissue-specific deletion of GDF11 or MSTN in conditional knockout mice.

## Developmental functions of GDF11 and MSTN

### Functions of MSTN/GDF11 in invertebrates

The physiological roles of ancestral MSTN/GDF11 in invertebrates, despite the availability of sequence information, are much less well known than those of GDF11 and MSTN in vertebrates. It should be noted that most of the invertebrate studies that utilized genetic mutations and RNA interference (RNAi) methods have provided evidence that ancestral *Mstn/Gdf11* positively regulates the development of diverse tissues and functions similarly to vertebrate *GDF11* rather than *MSTN* (Table 4). Ancestral *Mstn/Gdf11* was also shown to exhibit a broad expression pattern, which is similar to that of vertebrate *GDF11* but different from the muscle-specific expression pattern of vertebrate *MSTN*. For instance, *C. elegans daf-7*, a homolog of *GDF11* and *MSTN* expressed in ASI neurons, has been shown to promote the reproductive growth and development of worms^11,19,36^. Accordingly, *daf-7*-mutant worms exhibited a slower growth rate and increased dauer entry^11^. Genetic mutations or RNAi-mediated knockdown of *daf-7* also resulted in an increase in fat accumulation^18^, despite a reduction in the feeding rate, and declines in germ cell production and sperm motility^37,38^. However, there have been conflicting reports regarding the role of DAF-7 in the regulation of lifespan. While Shaw et al.^39^ reported that *daf-7* mutants and wild-type worms treated with *daf-7* RNAi exhibited an increased lifespan, Fletcher and Kim^12^ more recently demonstrated that DAF-7 promotes lifespan extension in response to dietary restriction and that age-dependent reduction in *daf-7* expression impairs the sensitivity of aged worms to the effects of dietary restriction on lifespan.

**Table 4:** Reported effects of GDF11 and MSTN on various tissues/cells of different animals.

Classification	Species	Growth factor	Physiological effects evaluated by			
			Endogenous gene knockdown/out		Application of recombinant proteins	
			Positive tissue effects	Negative tissue effects	Positive tissue effects	Negative tissue effects
Invertebrates (Ancestral gene, MSTN/GDF11)	Worm	DAF-7	• Increases lifespan^12^ • Promotes reproductive growth and development^19,36^ • Suppresses excess fat accumulation^18^ • Promotes gamete production and sperm function^37,38^	• Reduces lifespan^39^	NR	NR
	Insect	Myo	• Increases lifespan^16,42,147^ • Enhances muscle function and health^16^ • Promotes neuronal development and remodeling^13,40,41^ • Promotes normal molting and metamorphosis^45,46^ • Promotes disc growth^44^	• Reduces muscle size^43^ • Inhibits neuronal growth^43^	NR	NR
	Shrimp	MSTN/GDF11	• Increases growth rate^15,17^ • Improves survival rate^15^ • Promotes normal molting^47^	• Reduces muscle size^47^	NR	NR
Vertebrates (Independent genes, MSTN and GDF11)	Fish	Gdf11	NR	• Inhibits pancreas growth^148^	• Increases lifespan and antioxidant enzyme activity^68^	NR
		Mstn1/2	• Functions in immune defense^149,150^	• Inhibits skeletal muscle growth^149–152^	NR	• Inhibits skeletal muscle growth^153^
	Chicken	GDF11	• Promotes proper spinal cord patterning^154^	NR	NR	• Inhibits chondrogenesis and myogenesis^58^
		MSTN	NR	• Inhibits skeletal muscle growth^155,156^	• Promotes terminal differentiation of muscle progenitors^157^	• Inhibits skeletal muscle growth^158,159^
	Mouse/Rat	GDF11	• Promotes proper skeletal patterning^9,48^ • Promotes craniofacial development^48,50^ • Promotes temporal progression of neurogenesis^57^ • Promotes kidney development^53^ • Promotes pancreas development^54,160^ • Promotes spleen development^54^ • Promotes stomach development^54^ • Prevents left ventricular dilation^93^ • Attenuates liver fibrosis^161^ • Promotes bone development^26^ • Promotes chondrocyte maturation^26^ • Suppresses COPD^162^	• Inhibits neurogenesis^55,56,163^ • Induces PAH features^27^ • Induces oxidative stress^91^	• Rejuvenates cardiac tissue^84,85,164^ • Enhances skeletal muscle function and regeneration^67^ • Exerts neuroprotective effects^100–105,165,166^ • Improves vascularization^101,167^ • Improves skin health and repair^168–170^ • Improves kidney regeneration^171^ • Ameliorates colitis^172^ • Promotes bone/cartilage development^115,173^ • Protects against inflammatory arthritis^174^ • Improves metabolic homeostatsis^175–177^ • Improves endothelial function^178,179^ • Promotes blood antioxidant enzyme activities^180^	• Inhibits skeletal muscle growth/regeneration^70–73,90^ • Induces cachexia that leads to premature death/severe lethargy^90^ • Induces pathological hypertrophy in ventricular myocytes^89^ • Inhibits neurogenesis^56,106,107^ • Impairs liver regeneration^181,182^ • Induces kidney fibrosis/failure^183^ • Inhibits bone development and titanium implant healing^116–118^ • Inhibits chondrogenesis and callus formation^119^ • Induces myocardial cell death^90^ • Inhibits erythroid maturation^184^
		MSTN	Protects cardiac tissue^83^ • Protects joint and tendon^185^ • Promotes skin repair^186^	• Inhibits skeletal muscle growth/regeneration^4,35,187^ • Impairs cardiac function^78,79^ • Reduces lifespan^188^ • Inhibits axon growth^189^ • Inhibits bone development^26,113^ • Inhibits chondrogenesis^190^ • Impairs metabolism^191^	• Promotes tendon development and health^192^ • Promotes skin repair^170^ • Stimulates myoblast proliferation^63^ • Promotes neuron survival and neural outgrowth^98^ • Improves metabolic homeostasis^193^	• Inhibits skeletal muscle growth/regeneration^62,70,72^ • Inhibits neurogenesis^56^ • Inhibits bone development^112,194,195^ • Inhibits chondrogenesis^190^ • Impairs metabolic homeostasis^196^
	Human	GDF11	• Promotes proper orofacial development^51^ • Rejuvenates endothelial progenitor cells^197^	NR	• Enhances skin cell function^168^ • Promotes expansion of liver progenitor cells^161^ • Rejuvenates endothelial progenitor cells^197^	• Inhibits myoblast differentiation^70^ • Inhibits erythroid maturation^198^ • Induces PAH features^129^
		MSTN	NR	• Inhibits skeletal muscle development^7,187,199^	• Enhances ACL fibroblast function^200^ • Enhances muscle cell glucose uptake^201^	• Inhibits skeletal muscle growth^187^ • Inhibits bone development^202^

*ACL* anterior cruciate ligament, *COPD* chronic obstructive pulmonary disease, *GDF11* growth differentiation factor 11, *MSTN* myostatin, *Myo* myoglianin, *NR* not reported, *PAH* pulmonary arterial hypertension.

The insect gene *myo*, which is a homolog of *GDF11* and *MSTN*, is strongly expressed in muscle and glial cells and has been shown to promote neuronal development and remodeling^13,40,41^, prevent age-related muscular dysfunction^16^, and extend the lifespan in Drosophila^16,42^. Specifically, RNAi-mediated knockdown of *myo* in glia^13^ or muscle^16^ in Drosophila resulted in neuronal remodeling defects or exacerbated age-related climbing defects accompanied by premature death, respectively. Furthermore, a recent study suggested that Myo extends lifespan in flies by exerting protective functions in muscle homeostasis through regulating 26S proteasome function^42^. However, whether Myo regulates muscle mass in flies requires further investigation due to the existence of conflicting reports. As an illustration, while Demontis et al.^16^ showed no changes in muscle mass, body weight, and feeding behavior upon either muscle-specific Myo suppression or overexpression, Augustin et al.^43^ demonstrated that muscle-specific silencing of Myo increased larval weight and body-wall muscle size. More recently, Upadhyay et al.^44^ re-examined the same mutant flies and suggested that Myo deficiency did not result in larger muscles and that Myo is functionally distinct from vertebrate MSTN in terms of regulating muscle size. They also reported that Myo promotes imaginal disc growth in Drosophila^44^. Meanwhile, depletion of Myo through RNAi injection in third-instar cricket nymphs prevented the normal molting cycle and metamorphosis and led to reductions in nymph body size and weight, although the extended developmental period of the RNAi-treated nymphs eventually led them to exhibit a larger final insect size^45^. Injection of RNAi targeting *myo* into either fifth- or sixth-instar nymphs resulted in developmental arrest and death, highlighting the crucial role of Myo in promoting proper insect development^45^. Similar functions of Myo were also reported for cockroaches^46^.

In shrimp, the ancestral *Mstn*/*Gdf11* gene is expressed in diverse tissues, including muscle, hepatopancreas, eyestalk, heart, gill, and stomach, with the highest expression detected in the heart^17^. Endogenous expression of *Mstn*/*Gdf11* in shrimp muscle has been shown to peak immediately after molting, a period when significant growth occurs without restriction by a hard exoskeleton^17^. Interestingly, downregulation of the shrimp *Mstn*/*Gdf11* gene by tail-muscle injection of sequence-specific dsRNA led to a significantly impaired growth rate (68% reduction in final shrimp mass)^17^, an effect opposite to that observed after suppression of MSTN in vertebrates. Likewise, a separate study on shrimp revealed that silencing of the *Mstn*/*Gdf11* gene by tail-muscle injection of dsRNA significantly diminished growth and the survival rate^15^, indicating that ancestral MSTN/GDF11 in invertebrates is a positive regulator of growth and development, unlike vertebrate MSTN. Moreover, Zhuo et al.^47^ identified long (428 amino acids) and short (420 amino acids) forms of banana shrimp MSTN and FmMSTN, and the long form was positively correlated with a larger size in shrimp. Injection of dsRNA targeting *FmMstn* into these shrimp impaired their normal molting cycle but also resulted in the enlargement of the pleopod muscles^47^, which contradicts earlier findings. Further analysis and quantitation of the muscle fiber size in different muscle types are required to fully elucidate the effects of shrimp MSTN/GDF11 on controlling muscle development.

### Functions of GDF11 and MSTN during vertebrate development

During the embryonic development of vertebrates, *GDF11* and *MSTN* exhibit distinct expression patterns and functions. In mice, *Mstn* is initially expressed in the myotome compartment of somites at E9.5 and continues to be expressed in skeletal muscles to repress hyperplasia or increase the number of muscle fibers during development^4^. On the other hand, *Gdf11* is predominantly expressed in the mouse tail bud at E9.5 and specifies the positional identity of the skeleton along the anterior-posterior axis^9^. Correspondingly, *Gdf11*-null mice exhibit anterior homeotic transformations of the vertebrae by displaying an increase in the number of thoracic and lumbar vertebrae and vertebrosternal ribs^9,48^. It should be noted that *Gdf11* and *Mstn* double-mutants (*Mstn*^*−/−*^; *Gdf11*^*−/−*^) exhibited more extensive homeotic transformations of the axial skeleton than *Gdf11*-null mice, indicating that GDF11 and MSTN have some redundant functions related to the control of skeletal patterning^49^. GDF11 has also been shown to mediate proper craniofacial development, as *Gdf11*-null mice display high (60%) penetrance of cleft palate^48,50^. In further support of this role of GDF11, a recent study identified a family with orofacial clefting and vertebral/rib hypersegmentation harboring a dominant-negative missense mutation in GDF11, in which an arginine is substituted for a glutamine at the furin protease cleavage site (R298Q)^51^. Additional analysis confirmed that mutant GDF11 (R298Q) is not processed into the active form, indicating that this mutation is the underlying cause of the phenotypes observed in this family^51^. An earlier study reported that GDF11 also promotes tooth development and that electroporation-mediated transfer of the *Gdf11* gene to the amputated pulp of canine teeth enhances reparative dentin formation^52^. Furthermore, our group recently demonstrated that GDF11, in contrast to MSTN, facilitates osteogenesis during embryonic development and showed that compared to that in newborn wild-type mice, bone mass is diminished in newborn *Gdf11*-null mice and enhanced in newborn *Mstn*-null mice^26^.

Multiple studies that utilized *Gdf11*-null embryos demonstrated that GDF11 plays a crucial role in the development of various organs. Specifically, analysis of *Gdf11*-null mice revealed that the majority of these mice experience complete renal agenesis and failure of ureteric bud outgrowth from the Wolffian duct^53^. These mice were further shown to exhibit malformations of the stomach characterized by a two-fold reduction in the thickness of the gastric wall and a decreased number of gastric rugae (epithelial folds), a smaller spleen, and an abnormally shaped pancreas^54^, implying that GDF11 is essential for proper morphogenesis of the foregut-derived organs. *Gdf11* deficiency also resulted in the greater expansion of islet progenitor cells as well as the impairment of β-cell differentiation in the pancreas^54^. In the olfactory epithelium and retina, GDF11 was shown to inhibit neurogenesis by either repressing progenitor cell proliferation or altering progenitor cell fate^55,56^. This conclusion was supported by the significantly increased number of olfactory epithelium neurons and retinal ganglion cells in mice lacking GDF11 and the contrasting patterns observed in mice deficient in FST, an antagonist of GDF11. However, a delay in neuronal differentiation and gliogenesis was later reported in the spinal cord of *Gdf11*-null mice, suggesting that GDF11 promotes the temporal progression of neurogenesis in the developing spinal cord^57^. In addition, using chicken embryos, Gamer et al.^58^ demonstrated that implantation of beads soaked in human recombinant GDF11 protein into early wing buds led to a dramatic truncation of the limbs due to suppression of both myogenesis and chondrogenesis. In contrast, a recent analysis of *Gdf11*-null embryos at E15.5 and *Gdf11*-null sternal chondrocytes revealed that chondrocyte maturation was impaired under *Gdf11*-deficient conditions^26^. Moreover, skeletal muscle-specific deletion of *Gdf11* using conditional knockout techniques resulted in no differences in muscle mass and fiber type, indicating that the functions of GDF11 and MSTN in the skeletal muscles are most likely divergent^49^. Additional investigation of the skeletal muscles of *Gdf11*-null embryos will further clarify the role of GDF11 in myogenesis during development.

## Postnatal functions of GDF11 and MSTN in various tissues

### MSTN in skeletal muscle

The primary function of MSTN became evident when mice homozygous for *Mstn* deletion were shown to have a substantial increase in skeletal muscle mass, with individual muscle groups growing to approximately twice the normal size^4^. A significant increase in muscle mass was also observed in humans, cattle, sheep, and dogs with naturally occurring mutations in the *MSTN* gene^5–8^. Further analysis of *Mstn*-null mice revealed that MSTN inhibits both skeletal muscle fiber hyperplasia during early development and hypertrophy in adults. The direct role of MSTN in postnatal suppression of muscle fiber hypertrophy was demonstrated by the severe loss of muscle mass induced by systemic overexpression of MSTN in adult mice^35^ and the increase in muscle mass in adult mice treated with a monoclonal anti-MSTN antibody^59^. Paradoxically, circulating MSTN levels were shown to decrease with age in humans, implying that this decline is likely a secondary effect of age-related muscle loss^60^.

Previous studies that applied recombinant MSTN proteins have presented mixed results regarding the role of MSTN in satellite cells. For instance, while several early studies showed that treatment with recombinant MSTN proteins inhibited C2C12 myoblast proliferation^61,62^, Rodgers et al.^63^ more recently argued that recombinant MSTN proteins stimulate C2C12 proliferation, emphasizing that the source of the recombinant MSTN protein can impact the outcome of an experiment. Furthermore, primary myoblasts isolated from both *Mstn*-null mouse embryos and adult mice were shown to exhibit a significantly increased proliferation rate^64^, and skeletal muscle regeneration after toxin-induced injury was significantly enhanced in *Mstn*-null mice^65,66^, indicating that endogenous MSTN suppresses satellite cell proliferation, differentiation, and muscle regeneration.

### GDF11 in skeletal muscle

In contrast to *Mstn*-null mice, which survive to adulthood, *Gdf11*-null mice die shortly after birth, causing difficulties in identifying the role of GDF11 in adult tissue homeostasis. To overcome this limitation, Sinha et al.^67^ injected recombinant GDF11 proteins into aged mice and demonstrated that GDF11, in contrast to MSTN, acts as a rejuvenating factor in skeletal muscle. The aged mice treated with recombinant GDF11 proteins displayed striking improvements in muscle regeneration, exercise endurance, grip strength, myofibrillar and mitochondrial morphology, neuromuscular junctions, and the genomic integrity of muscle stem cells^67^. Furthermore, a more recent investigation in annual fish revealed that the application of GDF11 recombinant proteins boosted antioxidant enzyme activity in muscle, thus prolonging the lifespan^68^. *Gdf11* expression levels were also shown to increase in slow-twitch muscles of aged mice after 6 weeks of treadmill running^69^. However, multiple studies have failed to reproduce these results, showing that GDF11 is deleterious towards muscle repair. For example, Egerman et al.^70^ argued that treatment with recombinant GDF11 proteins significantly impaired muscle regeneration and satellite cell expansion in mice through a downstream signaling pathway identical to that utilized by MSTN. Likewise, Hinken et al.^71^ showed that the recombinant GDF11 protein repressed satellite cell expansion, while Hammers et al.^72^ demonstrated that recombinant GDF11 and MSTN proteins decreased the myotube diameter through the canonical SMAD2/3 pathway. Zhou et al.^73^ also observed that injection of recombinant GDF11 protein into older rats significantly hindered muscle regeneration and function and induced tissue fibrosis. In addition, several other recent studies have shown that exogenous GDF11 treatment inhibits muscle growth^74,75^ and reduces strength^76^, while overexpression of the GDF11 propeptide, which antagonizes both mature GDF11 and MSTN, exerts beneficial effects^23,77^.

Although most reports strongly suggest that exogenous GDF11 supplementation exerts an inhibitory effect on skeletal muscle growth and regeneration, further studies that employ genetic loss-of-function approaches are needed to fully elucidate the endogenous function of GDF11 in skeletal muscles. Interestingly, *Gdf11* expression levels were shown to peak in skeletal muscles in mice between 4 and 8 weeks of age, which is when the most dramatic postnatal muscle development occurs^20^. This expression pattern is similar to that observed in shrimp, in which the expression of *Mstn*/*Gdf11*, which was shown to promote growth unlike vertebrate MSTN, peaks immediately after molting^17^. Moreover, *Gdf11* expression levels were revealed to increase even further in *Mstn*-null mice during periods of rapid muscle growth^20^. To date, only one study has applied conditional knockout techniques in mice to investigate the postnatal functions of GDF11 in regulating skeletal muscle mass^49^. The study demonstrated that skeletal muscle-specific targeting of *Gdf11* had no significant effect on muscle mass, fiber number, or fiber type, demonstrating that GDF11 and MSTN exhibit distinct functions in controlling muscle size^49^. Additional examinations focusing on genetic approaches will further advance the understanding of the role of GDF11 in muscle biology.

### MSTN in heart

Despite its establishment as a potent inhibitor of skeletal muscle growth, MSTN has also been implicated in the regulation of cardiac tissue growth and function. A recent study that analyzed hearts of adult *Mstn*-null mice revealed that the absence of MSTN had no effect on heart weight but significantly decreased the end systolic diameter and increased fractional shortening^78^. Lim et al.^79^ consecutively demonstrated that *Mstn*-null mice subjected to ligation of the left anterior descending artery to induce myocardial infarction (MI) exhibited accelerated recovery of the ejection fraction, reduced cardiac fibrosis, and lower mortality, indicating that MSTN negatively affects cardiac function. Likewise, senescent MSTN-deficient mice were shown to display improved fractional shortening, smaller left ventricular diastolic and systolic diameters, and decreased cardiac fibrosis^80^, although an earlier study reported that MSTN has no significant effect on cardiac hypertrophy or fibrosis^81^. Additionally, transgenic mice overexpressing MSTN in cardiomyocytes exhibited interstitial fibrosis and impaired cardiac function^82^. Surprisingly, tamoxifen-induced, cardiomyocyte-specific deletion of *Mstn* in adult mice was reported to provoke severe cardiac hypertrophy and heart failure^83^. The authors of the study assumed that the much greater severity of the cardiac phenotype observed in the conditional knockout mice than in the straight knockout mice was due to the distinct modes of compensation^83^. To clarify the function of MSTN in hearts and whether the cardiac phenotypes of MSTN deficiency are influenced by the enhancement of skeletal muscle mass, additional examinations are needed.

### GDF11 in heart

In 2013, Loffredo et al.^84^ performed heterochronic parabiosis experiments in mice and identified GDF11 as a rejuvenating factor that circulates in plasma. The group utilized both proteomics (SOMAmer) and western blot analysis to determine that the circulating levels of GDF11 decline with age, reporting that restoration of youthful levels through daily intraperitoneal injections of recombinant GDF11 proteins reverses age-related cardiac hypertrophy^84^. Specifically, GDF11 rather than MSTN stimulated the dose-dependent inhibition of cardiac myocyte hypertrophy in vitro^84^. In further support of these results, Poggioli et al.^85^ showed that circulating GDF11/MSTN levels diminish with age in multiple mammalian species, and administration of recombinant GDF11 proteins dose-dependently decreased cardiac mass in both young and old mice after only 9 days. Likewise, Du et al.^86^ demonstrated that *Gdf11* expression levels decline in aged hearts and that either targeted myocardial delivery of the *Gdf11* gene or recombinant GDF11 protein enhanced cardiac function and effectively reduced infarct size after ischemic injury in aged mice, providing support for the anti-aging function of GDF11. The association of plasma GDF11/MSTN levels with cardiovascular outcomes and overall deaths in humans was also reported using SOMAmer technology, which revealed that in patients with stable ischemic heart disease, increased GDF11/MSTN levels were associated with decreased rates of cardiovascular events, left ventricular hypertrophy, and overall death^87^. Mechanistically, GDF11 was shown to increase intracellular calcium levels and activate SMAD2/3 to prevent cardiomyocyte hypertrophy^88^.

However, other groups have failed to observe the rejuvenating effects of GDF11 in cardiac tissues. After following the protocol used in a previous report^84^, Smith et al.^89^ showed that treatment of old mice with recombinant GDF11 proteins had no effect on cardiac mass, structure, or function. Moreover, recombinant GDF11 protein caused pathological hypertrophic signaling in neonatal rat ventricular myocytes, contradicting the classification of GDF11 as an anti-aging factor^89^. The same group subsequently published a dose-range study (0.5, 1.0, or 5.0 mg/kg) performed in young mice that underwent transverse aortic constriction (TAC) surgery and reported that although treatment with recombinant GDF11 proteins reduced pathological cardiac hypertrophy and fibrosis and improved cardiac function, the highest dose (5.0 mg/kg) led to severe cachexia and premature death, and they also issued a warning against the use of recombinant GDF11 proteins as a therapy^90^. Recombinant GDF11 protein treatment was also recently shown to increase the levels of reactive oxygen species in isoproterenol-treated H9C2 cells (rat heart-derived cardiomyoblast cell line)^91^ and impair cardiac function in old mice^75^. In addition, Egerman et al.^70^ pointed out that the SOMAmer and antibody used in the initial study by Loffredo et al.^84^ were nonspecific, claiming that circulating GDF11 levels actually increase with age and are a pro-aging factor. However, Poggioli et al.^85^ later proposed that the levels of GDF11 detected by Egerman et al.^70^ were in fact the levels of immunoglobulin light chain, generating further controversy regarding the circulating levels of GDF11. Applying a novel immunoplexed liquid chromatography with tandem mass spectrometry (LC-MS/MS) assay, Schafer et al.^60^ more accurately measured the circulating levels of GDF11 and reported that GDF11 levels remain constant in healthy adults throughout the lifespan. The study revealed that in older adults with severe aortic stenosis, higher GDF11 levels were associated with comorbidity and frailty^60^. Adding further controversy, a novel detection method using a parallel reaction monitoring (PRM) LC-MS/MS assay combined with immunoprecipitation recently showed that circulating levels of both GDF11 and MSTN significantly decline with age in female mice^92^.

In contrast to the large number of studies that investigated the effects of recombinant GDF11 proteins, only a single recent study has addressed the function of GDF11 based on cardiac-specific genetic deletion in mice. Using a *Myh6-Cre* transgene, Garbern et al.^93^ generated a conditional knockout mouse model in which *Gdf11* was targeted exclusively to cardiomyocytes and demonstrated that the mice exhibited progressive left ventricular dilation and a decrease in left ventricular systolic function at the age of 6 months. However, the authors also noted the adverse effects of the Cre recombinase itself and the possible compensatory expression of *Gdf11* in noncardiomyocytes, which prevented the clear interpretation of the mechanism underlying the above results^93^. Apparently, further genetic analysis with avoidance of Cre toxicity is required to delineate the endogenous role of GDF11 in cardiac tissues.

### MSTN in the brain

Despite the scarcity of information on the function of MSTN in the brain, a recent study showed that *Mstn* is broadly expressed throughout the adult rat central nervous system, including most neurons, axons, oligodendrocytes, astrocytes, and ependymal cells, suggesting that MSTN may play a crucial role in the brain^94^. Regarding the role of MSTN in the nervous system, examination of adult *Mstn*-null mice revealed that these mice exhibit increases in the number and size of axons and a delay in their age-related reduction^95^. Furthermore, MSTN-deficient mice were shown to display enhanced myelin thickness in motor axons and an increase in the number of sensory axons^96^. These mice were also shown to present a smaller brain size than wild-type mice at the age of 4 months, but the mechanism of brain size regulation by MSTN remains unclear^97^. Meanwhile, conflicting reports exist regarding the effects of recombinant MSTN proteins on neuronal cells. For instance, while Kerrison et al.^98^ showed that recombinant MSTN proteins dose-dependently enhanced the survival of retinal ganglion cells and neurite outgrowth, others demonstrated that recombinant MSTN proteins decreased the formation of neuronal colonies^56^ or suggested that MSTN inhibits neurogenesis in the olfactory system^99^.

### GDF11 in the brain

The perinatal lethality observed in *Gdf11*-deficient mice has led to multiple studies that investigated the effects of recombinant GDF11 proteins on adult neurogenesis, demonstrating that GDF11 is a pro-neurogenic and pro-angiogenic factor. Shortly after Loffredo et al.^84^ reported GDF11 as a rejuvenating agent that protects the aged heart, the same group proposed that GDF11 exerts anti-aging effects on the brain, which was supported by the improvement of the cerebral vasculature and the enhancement of neurogenesis after the treatment of old mice with recombinant GDF11 proteins^100^. A separate experiment also showed that systemic delivery of recombinant GDF11 proteins enhanced hippocampal neurogenesis and vasculature in old mice by acting on brain endothelial cells, and only GDF11 but not MSTN promoted VEGF secretion in brain endothelial cells^101^. Likewise, a single injection of recombinant GDF11 protein was shown to improve short-term visual memory in middle-aged mice through upregulation of SOX2 expression^102^. Furthermore, treatment with recombinant GDF11 proteins was shown to promote neurogenesis and angiogenesis in mouse and rat models of stroke^103,104^ and in a mouse model of Alzheimer’s disease, revealing GDF11 as a potential therapeutic option for neurodegenerative disorders^105^. In contrast, in vitro data on the effects of recombinant GDF11 protein exposure to neural stem cell lines demonstrated that GDF11 suppresses cell proliferation and migration, suggesting that GDF11 should be a target for pharmacological blockade^106,107^. While the majority of studies presented the beneficial effects of recombinant GDF11 treatment on the mature nervous system, additional analysis utilizing genetic knockdown or conditional knockout of *Gdf11* will further advance the understanding of the role and action mechanism of endogenous GDF11 in the adult brain.

### MSTN in bone

The deficiency of MSTN has been described to result in not only an enlargement in skeletal muscle mass but also an increase in bone mass. In this regard, *Mstn*-null mice were shown to exhibit enhanced bone mineral density in various parts of the skeleton^26,80,108^. In humans, genetic polymorphisms in *MSTN* were demonstrated to be associated with peak bone mineral density^109^. The effects of MSTN on bone may be both direct and indirect through the influence of skeletal muscle. While the indirect positive effect of enhanced skeletal muscle mass on bone strength was evidenced in *Mstn*-null mice^110^, the direct inhibition of osteoblast differentiation and stimulation of osteoclast formation by MSTN were also reported^26,111–114^. Surprisingly, despite the relatively low expression of *Mstn* in primary mouse osteoblasts and osteoclast precursors under physiological conditions, siRNA-mediated knockdown or genetic knockout of *Mstn* noticeably altered the differentiation rate of these cells^26,113^, highlighting the significant role of MSTN in the direct regulation of bone cells.

### GDF11 in bone

As opposed to the consistent reports on the inhibitory function of MSTN on osteogenesis, the reports of the effects of GDF11 on adult bone homeostasis are controversial. In 2015, Zhang et al.^115^ demonstrated that circulating GDF11 levels were significantly diminished in both aged humans and patients with osteoporosis, and *Gdf11* expression levels were substantially downregulated in the bone marrow of aged mice and mice with osteoporosis. Additionally, the group showed that treatment with recombinant GDF11 proteins significantly promoted osteoblast differentiation and inhibited adipogenesis of bone marrow mesenchymal stem cells^115^, emphasizing the pro-osteogenic role of GDF11, which is in contrast with the function of MSTN. However, Lu et al.^116^ subsequently published results showing the opposite results, indicating that recombinant GDF11 proteins inhibited osteoblast differentiation of bone marrow mesenchymal stem cells through a downstream signaling pathway identical to that of MSTN and that injection of recombinant GDF11 proteins suppressed bone formation in mice. In the same year, Liu et al.^117^ also reported similar findings and demonstrated that recombinant GDF11 treatment led to bone loss in both young and aged mice through impairment of osteoblast differentiation and increased osteoclast formation. Later, the same group further showed that recombinant GDF11 negatively affects bone healing in mice^118,119^. Moreover, in postmenopausal women, increased levels of circulating GDF11 were associated with decreased bone mineral density, demonstrating the inhibitory effect of GDF11 on bone formation^120^.

In an attempt to avoid the controversy surrounding the effects of recombinant GDF11 proteins, our group has recently applied conditional knockout strategies in mice to examine the endogenous function of GDF11 in osteogenesis^26^. Our findings revealed that both time-specific ubiquitous deletion and limb mesenchyme-specific deletion of *Gdf11* resulted in diminished bone mass in young adult mice, suggesting that GDF11 endogenously promotes bone development, in contrast to MSTN^26^. Furthermore, both *Gdf11-*null osteoblasts and wild-type osteoblasts subjected to siRNA-mediated *Gdf11* knockdown exhibited impaired differentiation and mineralization, while the opposite effects were observed in wild-type osteoblasts transfected with full-length *GDF11* cDNA^26^. Whether there is a difference in the cellular and physiological outcomes of treatment with the full-length cDNA and mature forms of the GDF11 protein requires further investigation. A previous study demonstrated that skeleton-specific transgenic overexpression of the GDF11 propeptide, which is capable of inhibiting both mature forms of GDF11 and MSTN, enhanced bone formation in mice during embryogenesis and postnatal development^121^. However, these mice were shown to exhibit a posterior homeotic transformation in the cervical vertebra, resulting in transformation of the seventh cervical vertebra into a thoracic vertebra, which is the exact opposite of the anterior homeotic transformations observed in *Gdf11*-null mice^9,48^. Furthermore, the authors did not provide information on the relative expression of endogenous *Gdf11* and the transgene during developmental stages, limiting the potential for the clear interpretation of the results^122^. Additional examination focused on genetic studies in mice will lead to additional insights into the endogenous mechanism of action of GDF11 in postnatal bone remodeling.

## Therapeutic implications of GDF11 activation

### Recombinant GDF11 protein as a therapeutic option

After the initial reports of GDF11 as a rejuvenating agent for the heart, skeletal muscle, and brain, numerous groups have evaluated the effects of recombinant GDF11 protein administration on various tissues (Table 5). However, despite using similar treatment and dosage regimens, multiple groups have produced widely varying results, especially in skeletal muscle and heart. Regarding this issue, Poggioli et al.^85^ pointed out the existence of batch-to-batch variations in the concentrations of recombinant GDF11 proteins, which was also confirmed by the manufacturer, and suggested that differences in protein sources, protein refolding efficiencies, and protein concentrations all possibly contributed to the disparity in the outcomes. Likewise, Rodgers and Eldridge^34^ highlighted the possible significant influence of the source and quality of recombinant proteins on experimental results and indicated that both Sinha et al.^67^ and Egerman et al.^70^, who reported opposing results, utilized bacterially generated recombinant proteins that may be less effective or even produce different effects depending on the folding status. In support of this claim, Rodgers et al.^63^ demonstrated that recombinant MSTN proteins produced in bacteria and eukaryotes behave differently in the regulation of C2C12 myoblast proliferation. Most importantly, even though differences in the type 1 receptor-binding residues and signaling potency between mature GDF11 and MSTN have been reported^25^, it is difficult to rule out the fact that recombinant GDF11 and MSTN proteins cannot be effectively distinguished due to their high sequence similarity, revealing the possibility that the responses mediated by recombinant GDF11 protein treatment actually reflect the endogenous functions of MSTN. Apparently, due to the large discrepancy in the reported effects of recombinant GDF11 protein treatment, further establishment of experimental settings that generate more reliable outcomes as well as different strategies for GDF11 supplementation will be needed for further consideration of GDF11 as a therapeutic option.

**Table 5:** The effects of in vivo recombinant GDF11 protein treatment on skeletal muscle, heart, brain, and bone.

Tissue	Study (year of publication)	rGDF11 dosage (mg/BW)	Treatment duration (once-daily)	Route of administration	Product source	Model type	Physiological effects		
							Beneficial	No effect	Harmful
Skeletal muscle	Sinha et al. (2014)^67^	0.1 mg/kg	4–5 weeks	IP	PeproTech	Young (2- to 3-month-old) and old (22- to 24-month-old) mice	O		
	Egerman et al. (2015)^70^	0.1 mg/kg	5 weeks	IP	R&D Systems	Old mice (23-month-old)		O	
		0.3 mg/kg	17 days	IP	R&D Systems	Young mice (16-week-old)			O
	Zhou et al. (2017)^73^	0.1 mg/kg	6 weeks	IP	R&D Systems	Rat model of skeletal muscle injury (10- to 12-month-old)			O
	Harper et al. (2018)^90^	5.0 mg/kg	2 weeks	IP	R&D Systems	Mice that underwent TAC surgery (12- to 13-week-old)			O
	Zhou et al. (2019)^68^	0.2 mg/kg	3 and 7 weeks	Oral (rGDF11-soaked food)	Self-produced	Annual fish (9-month-old)	O		
	Roh et al. (2019)^75^	0.1 mg/kg	4 weeks	IP	PeproTech	Old mice (24-month-old)			O
Heart	Loffredo et al. (2013)^84^	0.1 mg/kg	4 weeks	IP	PeproTech	Young (2-month-old) and old (21- to 23-month-old)	O		
	Smith et al. (2015)^89^	0.1 mg/kg	4 weeks	IP	R&D Systems	Old mice (24-month-old)		O	
	Poggioli et al. (2016)^85^	0.5 mg/kg	9 days	IP	PeproTech	Young (2-month-old) and old (22-month-old) mice	O		
		1.0 mg/kg	9 days	IP	PeproTech		O		
	Du et al. (2017)^86^	0.1 mg/kg	25 days	IP	R&D Systems	Young (3-month-old) and old (21-month-old) mice after I/R injury	O		
	Harper et al. (2018)^90^	0.5 mg/kg	2 weeks	IP	R&D Systems	Mice that underwent TAC surgery (12- to 13-week-old)	O		
		1.0 mg/kg	2 weeks	IP	R&D Systems		O		
		5.0 mg/kg	2 weeks	IP	R&D Systems		O		
	Roh et al. (2019)^75^	0.1 mg/kg	4 weeks	IP	PeproTech	Old mice (24-month-old)			O
Brain	Katsimpardi et al. (2014)^100^	0.1 mg/kg	4 weeks	IP	PeproTech	Old mice (21- to 23- month-old)	O		
	Zhang et al. (2018)^105^	0.1 mg/kg	4 weeks	IV	Abnova	Mouse model of Alzheimer’s disease (12-month-old)	O		
	Zhang et al. (2018)^102^	0.1 mg/kg	1 day	IP	PeproTech	Young (1.5-month-old) and middle-age (9-month-old) mice	O		
	Ma et al. (2018)^103^	0.01 mg/kg	7 days	IV	PeproTech	Rat model of stroke (42- to 48-day-old)		O	
		0.03 mg/kg	7 days	IV	PeproTech			O	
		0.1 mg/kg	7 days	IV	PeproTech		O		
		0.2 mg/kg	7 days	IV	PeproTech		O		
	Lu et al. (2018)^104^	0.1 mg/kg	7–13 days after stroke	IP	PeproTech	Mouse model of stroke (8- to 10-week-old)	O		
	Ozek et al. (2018)^101^	1.0 mg/kg	4 weeks	IP	PeproTech	Young (2- to 3-month-old) and old (22- to 23-month-old) mice	O		
Bone	Lu et al. (2016)^116^	0.1 mg/kg	12 weeks	IP	R&D Systems	Middle-age mice (12-month-old)			O
	Liu et al. (2016)^117^	0.1 mg/kg	6 weeks	IP	PeproTech	Young (9-week-old) and old (18-month-old) mice			O
		0.3 mg/kg	6 weeks	IP	PeproTech	Old mice (18-month-old)			O
	Zheng et al. (2019)^119^	0.1 mg/kg	10 or 21 days	IP	PeproTech	Mouse model of femur fracture (12-week-old)			O
	Liu et al. (2020)^118^	0.1 mg/kg	6 weeks	IP	PeproTech	Young (2-month-old) and old (18-month-old) mice			O

*BW* body weight, *IP* intraperitoneal, *I/R* ischemia-reperfusion, *IV* intravenous, *rGDF11* recombinant growth differentiation factor 11, *TAC* transverse aortic constriction.

### Potential adverse effects of targeting GDF11

Ample studies that noted the detrimental effects of recombinant GDF11 protein injection have also indicated GDF11 as a potential target for pharmacological blockade. However, the relatively little information is available on the endogenous functions of GDF11 in regulating adult physiology, which indicates the need for a cautious approach in the development of GDF11 inhibitors. In fact, sotatercept (ACE-011), an ACVR2A fusion protein originally designed by Acceleron Pharma to increase bone mineral density^123^, unexpectedly promoted rapid increases in hematocrit, hemoglobin, red blood cells, and late-stage erythropoiesis, which were suggested to be caused by suppression of endogenous GDF11^124^, although recent studies using conditional knockout techniques refuted this mechanism^125,126^. In addition, our group has recently demonstrated that transgenic overexpression of FST, an endogenous inhibitor of MSTN, GDF11, and activins, substantially enhanced muscle mass but induced spontaneous tibial fractures due to a reduction in bone mineral density, implying that inhibition of GDF11 may have adverse effects on bone^26^. Therefore, possible side effects triggered by both exogenous administration and endogenous inhibition of GDF11 and the means to resolve them should be evaluated with caution in order to enhance the potential for GDF11 to be applied in clinical settings.

### Conclusion and future perspectives

The remarkable sequence similarity between GDF11 and MSTN led to the assumption that the two molecules are functionally redundant. However, multiple genetic studies in mice provide clear evidence that they play distinct roles under a range of physiological conditions. Notably, the perinatal lethality observed in *Gdf11*-null mice, in contrast to the long-term viability of MSTN-deficient mice, led to complications in characterizing the role of GDF11 in adult tissues and caused many groups to utilize recombinant GDF11 proteins to identify its postnatal function. However, difficulties in biochemically distinguishing between the recombinant GDF11 and MSTN proteins as well as variations in the quality of recombinant proteins aroused much controversy regarding the effects of GDF11 treatment. Indeed, while numerous studies have presented the beneficial physiological outcomes after supplementation with recombinant GDF11 proteins, providing a rationale for its therapeutic application, an equally large number of studies have also underscored its harmfulness, demonstrating GDF11 as a potential therapeutic target for inhibition. Therefore, future studies should focus on implementing genetic knockdown or conditional knockout techniques, which may be more promising approaches to differentiate the endogenous functions of GDF11 and MSTN and their regulatory mechanisms. Furthermore, reliable research strategies to improve the consistency of test results are needed to support the progression of GDF11 therapy or GDF11 inhibitors to clinical trials.

## Supplementary information

Table S1
