# Supplementary material for: Similar sequences but dissimilar biological functions of GDF11 and myostatin
Source: Exp Mol Med. 2020 Oct 19;52(10):1673–93. doi: 10.1038/s12276-020-00516-4 (PMC8080601; doi:10.1038/s12276-020-00516-4)
Supplement: Supplementary file 1 — Table S1 [file 12276_2020_516_MOESM1_ESM.docx]

**Table S1. List of vertebrates analyzed using Aminode in Fig. 2a.**

| Protein | Species |
| --- | --- |
| GDF11 | *Homo sapiens, Pan troglodytes, Chlorocebus sabaeus, Rattus norvegicus, Mus musculus, Pteropus vampyrus, Felis catus, Dasypus novemcinctus, Sus scrofa, Callithrix jacchus, Gorilla gorilla, Oryctolagus cuniculus, Mustela putorius furo, Ochotona princeps, Bos Taurus, Sarcophilus harrisii, Erinaceus europaeus, Anolis carolinensis, Oreochromis niloticus, Gasterosteus aculeatus, Takifugu rubripes, Oryzias latipes, Xiphophorus maculatus, Tetraodon nigroviridis, Gadus morhua, Lepisosteus oculatus, Danio rerio, Astyanax mexicanus, Latimeria chalumnae* |
| MSTN | *Homo sapiens, Papio anubis, Chlorocebus sabaeus, Macaca mulatta, Pan troglodytes, Gorilla gorilla, Pongo abelii, Tarsius syrichta, Tursiops truncates, Sus scrofa, Felis catus, Ailuropoda melanoleuca, Oryctolagus cuniculus, Ictidomys tridecemlineatus, Otolemur garnettii, Loxodonta africana, Equus caballus, Cavia porcellus, Dasypus novemcinctus, Canis familiaris, Pteropus vampyrus, Sarcophilus harrisii, Monodelphis domestica, Echinops telfairi, Mustela putorius furo, Rattus norvegicus, Mus musculus, Erinaceus europaeus, Sorex araneus, Meleagris gallopavo, Anas platyrhynchos, Taeniopygia guttata, Ficedula albicollis, Anolis carolinensis, Macropus eugenii, Pelodiscus sinensis, Ochotona princeps* |
| INHBA | *Homo sapiens, Pan troglodytes, Gorilla gorilla, Pongo abelii, Nomascus leucogenys, Papio anubis, Macaca mulatta, Chlorocebus sabaeus, Otolemur garnettii, Callithrix jacchus, Bos taurus, Ovis aries, Rattus norvegicus, Mus musculus, Ochotona princeps, Tupaia belangeri, Dipodomys ordii, Equus caballus, Pteropus vampyrus, Myotis lucifugus, Canis familiaris, Mustela putorius furo, Ailuropoda melanoleuca, Erinaceus europaeus, Ornithorhynchus anatinus, Sus scrofa, Tursiops truncatus, Tarsius syrichta, Procavia capensis, Macropus eugenii, Monodelphis domestica, Meleagris gallopavo, Gallus gallus, Taeniopygia guttata, Ficedula albicollis, Pelodiscus sinensis, Anolis carolinensis, Echinops telfairi, Xenopus tropicalis* |
| INHBB | *Homo sapiens, Gorilla gorilla, Otolemur garnettii, Myotis lucifugus, Rattus norvegicus, Mus musculus, Tursiops truncatus, Bos taurus, Loxodonta africana, Dipodomys ordii, Papio anubis, Sarcophilus harrisii, Cavia porcellus, Macaca mulatta, Ictidomys tridecemlineatus, Anolis carolinensis, Ochotona princeps, Latimeria chalumnae, Oreochromis niloticus, Tetraodon nigroviridis, Takifugu rubripes, Xiphophorus maculatus, Danio rerio, Astyanax mexicanus, Lepisosteus oculatus, Poecilia formosa, Ficedula albicollis, Gasterosteus aculeatus* |
| TGFB1 | *Homo sapiens, Pan troglodytes, Papio anubis, Chlorocebus sabaeus, Nomascus leucogenys, Mustela putorius furo, Felis catus, Microcebus murinus, Ovis aries, Equus caballus, Rattus norvegicus, Mus musculus, Ictidomys tridecemlineatus, Loxodonta africana, Ochotona princeps, Cavia porcellus, Otolemur garnettii, Dipodomys ordii, Dasypus novemcinctus, Erinaceus europaeus, Macropus eugenii, Monodelphis domestica, Pteropus vampyrus, Anolis carolinensis, Latimeria chalumnae, Xenopus tropicalis* |
| TGFB2 | *Homo sapiens, Papio anubis, Nomascus leucogenys, Macaca mulatta, Pan troglodytes, Equus caballus, Canis familiaris, Ailuropoda melanoleuca, Felis catus, Callithrix jacchus, Gorilla gorilla, Myotis lucifugus, Oryctolagus cuniculus, Tupaia belangeri, Dipodomys ordii, Dasypus novemcinctus, Choloepus hoffmanni, Otolemur garnettii, Rattus norvegicus, Mus musculus, Ochotona princeps, Pteropus vampyrus, Mustela putorius furo, Macropus eugenii, Tarsius syrichta, Sarcophilus harrisii, Monodelphis domestica, Echinops telfairi, Erinaceus europaeus, Gallus gallus, Ficedula albicollis, Taeniopygia guttata, Pelodiscus sinensis, Xenopus tropicalis, Latimeria chalumnae, Procavia capensis, Oreochromis niloticus, Gasterosteus aculeatus, Gadus morhua, Danio rerio, Tetraodon nigroviridis, Lepisosteus oculatus, Takifugu rubripes* |
| TGFB3 | *Homo sapiens, Nomascus leucogenys, Pan troglodytes, Gorilla gorilla, Pongo abelii, Macaca mulatta, Chlorocebus sabaeus, Papio anubis, Equus caballus, Loxodonta africana, Cavia porcellus, Ictidomys tridecemlineatus, Microcebus murinus, Otolemur garnettii, Callithrix jacchus, Rattus norvegicus, Mus musculus, Dipodomys ordii, Mustela putorius furo, Ailuropoda melanoleuca, Felis catus, Monodelphis domestica, Canis familiaris, Dasypus novemcinctus, Pelodiscus sinensis, Ficedula albicollis, Tupaia belangeri, Choloepus hoffmanni* |

GDF11, growth differentiation factor 11; INHBA, inhibin subunit beta A; INHBB, inhibin subunit beta B; MSTN, myostatin; TGFB, transforming growth factor beta.
